# Transgenerational response to early spring warming in *Daphnia*

**DOI:** 10.1038/s41598-019-40946-3

**Published:** 2019-03-14

**Authors:** Kenji Toyota, Maria Cambronero Cuenca, Vignesh Dhandapani, Antonio Suppa, Valeria Rossi, John K. Colbourne, Luisa Orsini

**Affiliations:** 1 Environmental Genomics Group, School of Biosciences, University of Birmingham, Birmingham, B15 2TT UK; 2 Department of Biological Science, Faculty of Science, Kanagawa University, Hiratsuka, Kanagawa 259-1293 Japan; 3 Department of Biological Science and Technology, Tokyo University of Science, Katsushika, Tokyo Japan; 4 Department of Chemistry, Life Sciences and Environmental Sustainability University of Parma, Department of Life Sciences, Viale Usberti, 11/A, Parma, Italy; 5 Present Address: Aquatic Ecology Department, EAWAG, Kastanienbaum, Switzerland

## Abstract

Temperature and photoperiod regulate key fitness traits in plants and animals. However, with temperature increase due to global warming, temperature cue thresholds are experienced at shorter photoperiods, disrupting the optimal seasonal timing of physiological, developmental and reproductive events in many species. Understanding the mechanisms of adaptation to the asynchrony between temperature and photoperiod is key to inform our understanding of how species will respond to global warming. Here, we studied the transgenerational mechanisms of responses of the cyclical parthenogen *Daphnia magna* to different photoperiod lengths co-occurring with warm temperature thereby assessing the impact of earlier spring warming on its fitness. *Daphnia* uses temperature and photoperiod cues to time dormancy, and to switch between sexual and asexual reproduction. *Daphnia* life cycle offers the opportunity to measure the relative contribution of plastic and genetic responses to environmental change across generations and over evolutionary time. We use transgenerational common garden experiments on three populations ‘resurrected’ from a biological archive experiencing temperature increase over five decades. Our results suggest that response to early spring warming evolved underpinned by a complex interaction between plastic and genetic mechanisms while a positive maternal contribution at matching environments between parental and offspring generation was also observed.

## Introduction

Organisms respond to changing environments by shifting their distribution, via genetic adaptation or through plasticity of fitness related traits^1^. Plasticity is expected to play a key role in rapidly changing environments, especially when genetic adaptation may be constrained under predicted global change^2,3^. So far, within-generation plasticity (WGP) is advocated as the main mechanism of response to environmental change, allowing for rapid adjustments to novel environmental conditions^4–6^. Yet this consensus is subject to a scarcity of studies on the mechanisms of genetic adaptation to environmental change, because of the significant challenges at identifying the genetic elements underpinning adaptive phenotypic trait variation in nature^1,3,7^. Moreover, it is unclear how plasticity impacts long-term evolutionary responses to environmental change, as plasticity can either help^8,9^ or hinder^10^ evolutionary responses. Obtaining evidence that identifies the relative contribution of genetic adaptation and plasticity in the wild represents an ongoing challenge^6^.

Plasticity experienced within a generation may influence the response of following generations via non-genetic or epigenetic processes. This form of plasticity is known as transgenerational plasticity (TGP) and occurs when the environment that individuals experience influences the expression of traits in their offspring^11–13^. TGP is especially relevant to understand the impact of environmental change that persists across generations. Currently, the term TGP is loosely used to include any non-genetic effects that are observed in the offspring generation, which is associated with exposure of a previous generation to a new environmental condition^14^. However, TGP may also be adaptive, enhancing offspring performance, by buffering populations against the immediate effects of environmental change and providing time for genetic adaptation to happen in the longer term^15^. Adaptive transgenerational plasticity, mediated by parental effects, maximizes offspring fitness when the parental and the offspring environments are matched^12,13^. Examples of adaptive transgenerational plasticity include the effect of maternal environment on the timing of seed germination^16^ and shade-avoidance in plants^17^. Understanding the role of adaptive transgenerational plasticity is key to predict the consequences of parental effects on population dynamics, and to inform our understanding of how species will respond to rapid environmental change^18^. Presently, the prevalence and strength of TGP in natural systems remains controversial^13^.

Temperature and photoperiod are two pivotal cues that regulate key fitness traits in plants and animals (e.g. flowering time and dormancy). However, whereas climate is changing, photoperiod is stable across latitudes^19,20^. The asynchronous change of temperature and photoperiod cues has disrupted the optimal seasonal timing of physiological, developmental and reproductive events in many species^21–23^. Therefore, many organisms have experienced a decline in fitness when preparing to develop, reproduce, hibernate, enter dormancy, or migrate at seasonally inappropriate times^22^. Parthenogenetic zooplankton species (e.g. daphniids, rotifers) use both temperature and photoperiod cues to time life history events such as male production and dormancy^21,24^. Generally, short photoperiod and low temperatures induce a switch from the production of clonal (parthenogenetic) females to the parthenogenetic generation of males and to initiating sexual reproduction, which culminates in dormant embryos^25^. Conversely, long photoperiod and high temperatures are cues for the termination of dormancy in spring^21,22^. Dormant embryos are early stage embryos that arrest their development to overwinter; dormant embryos at the water-sediment interface hatch during the following growing season contributing to the genetic diversity of the local population^26–28^. A proportion of these embryos becomes buried in the sediment missing the opportunity to hatch thereby creating biological archives, which represent an excellent resource to investigate population dynamics in natural systems over evolutionary time^29–31^. Because of the changes in water temperature due to global warming, the temperature cue thresholds are experienced by some parthenogenetic zooplankers at shorter photoperiods altering population-level investment in dormancy and male formation^32,33^. However, the impact of persistent asynchrony between temperature and photoperiod across generations is poorly understood. Understanding the transgenerational impact of this asynchrony is helpful to predict how rapidly these species, and the community which they sustain, may track climatic change.

Here, we study the impact of the asynchrony between temperature and photoperiod on the fitness of the freshwater crustacean *Daphnia magna. Daphnia* are keystone species central to the food-web of lentic freshwater ecosystems worldwide^34,35^. We study within-generation plastic and genetic responses, as well as the cross-generational responses of fitness-linked life history traits to environmental conditions that mimic early spring warming (warm temperature and short photoperiod) as compared to a typical spring environment (warm temperature and long photoperiod).

Studies that investigate mechanisms of response to environmental change face two main limitations. If genetically diverse species are studied, the same genotype or a diversity of genotypes cannot be replicated across environmental exposures and transgenerational responses to environmental change are confounded by genetic variation. Conversely, if clonal species are used and genotypes are replicated across experiments, genetic diversity is not representative of sexually reproducing species. *Daphnia*’s life cycle overcomes these limitations to reveal the relative contribution of plastic and genetic responses to environmental change across generations and over extended time periods^36,37^. *Daphnia* alternates sexual recombination with asexual (clonal) reproduction. Sexual recombination results in early stage embryos that can be ‘resurrected’ and propagated indefinitely as clonal lineages in the laboratory^38^. These properties and a short life cycle provide the advantages of clonal species while retaining the natural genetic diversity. Taking full advantage of these properties, we measured transgenerational fitness changes in three populations of dormant embryos previously resurrected from the biological archive of Lake Ring and spanning 50 years^39^. We quantified WGP and TGP as changes in trait values of fitness-linked life history traits between environments and across generations. We quantified genetic responses to photoperiod as mean trait differences among populations and as genetic divergence at 15 candidate genes, previously linked to environmentally driven local adaptation^40,41^. We also performed a gene-traits association analysis between the life history traits measured in the parental generation and the 15 candidate genes in an effort to identify the genetic basis of fitness-linked life history traits responding to photoperiod and temperature cues. Our study provides important insights into the transgenerational mechanisms of response to early spring warming in a freshwater keystone species.

## Methods

### Experimental design

We investigate the impact of temperature and photoperiod cues on three populations of *D. magna* separated in time and previously revived from a biological archive of Lake Ring, a shallow mixed lake (maximum depth is 5 m) in Jutland, Denmark (55°57′51.83″N, 9°35′46.87″E)^42^. According to historical records^43^ and paleolimnological analyses^30,36,38^, the lake experienced a steadily, even if modest (∼1 °C), increase in temperature over the last five decades^36^. Further, changes in water chemistry and transparency occurred over time because of eutrophication and agricultural land use. These changes are described in detail elsewhere^39^ and summarized in Supporting Information Data 1. The populations are hereafter referred to as: P1: population resurrected from layers of sediment dated >1999; P2: population resurrected from layers of sediment dated 1975–1985; P3: population resurrected from layers of sediment dated 1960–1970.

**Figure 1:**
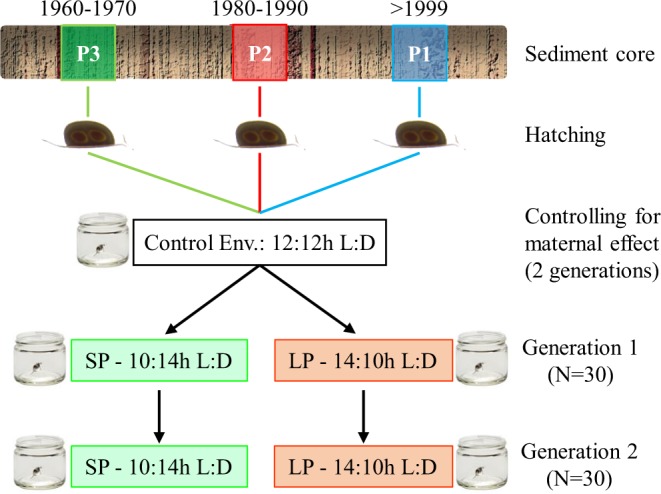
Experimental design. *Daphnia magna* dormant embryos previously revived from three populations separated in are: P1 (blue): population resurrected from layers of sediment dated > 1999; P2 (red): population resurrected from layers of sediment dated 1975–1985; P3 (green): population resurrected from layers of sediment dated 1960–1970. Clonal lineages established from the resurrected genotypes were maintained in common garden conditions for two generations to reduce interference from maternal and grandmaternal effect. After two generations in common garden conditions, two randomly selected genetically identical copies of each genotype from the second or following broods are assigned to either long photoperiod (LP; 14:10 h light: dark regime, orange) or short photoperiod (SP; 10:14 h light: dark regime, green) for two generations (G1 and G2). A suite of life history traits is measured in both generations.

*Daphnia* reproduces parthenogenetically in the laboratory, allowing experiments across generations. Several parthenogenetic broods are produced in each generation (up to eight) allowing the same genotype to be used in parallel experiments. Between nine and eleven genotypes per population (Table S1; N = 30) were used in a common garden experiment over two parthenogenetic generations (Fig. 1). The sample size per population was chosen based on a previous study that determined the threshold sample size required to assess genetic diversity in *Daphnia* populations^30^. According to this study, 10–15 genotypes capture the genetic diversity of natural populations both in space and time.

Prior to the experiment, clonal lines established from the thirty genotypes were maintained for two generations in common garden conditions (20 °C, 12:12 h light: dark regime; fed *ad libitum* with 0.8 mg Carbon/L of *Chlorella vulgaris* CCAP strain no. 211/11B) to reduce potentially confounding maternal and grandmaternal effects (Fig. 1). Individual juveniles of 24 h from the second or following broods were randomly selected to establish experimental generations and assigned to experimental conditions. Different broods from the same generation were used to ensure developmental synchrony among clonal lineages in the experiment. Experimental clonal lineages were kept in individual jars of 100 ml, filled with filtered borehole water, which was refreshed every second day, and fed daily 0.8 mg Carbon/L of *Chlorella vulgaris*.

After two generations in common garden conditions, 24 h old individual juveniles from the second or following broods were randomly assigned to the experimental conditions for two generations (G1, G2): short photoperiod (SP; 10:14 h light: dark regime) and long photoperiod (LP; 14:10 h light: dark regime) (Fig. 1). The temperature for both photoperiods was 20 °C, so that changes in photoperiod could be studied in a typical spring environment (warm temperature and long photoperiod), acting as a control, and in conditions that mimicked early spring warming (warm temperature and short photoperiod).

The following fitness-linked life history traits were measured in G1 and G2: size at maturity (the distance between the head and the base of the tail spine), age at maturity (first time parthenogenetic eggs are released in the brood pouch), fecundity (number of juveniles across 8 broods), interval between broods (time interval between each pair of broods averaged across eight broods), proportion of male offspring across 8 broods, and mortality. For size at maturity, all animals were measured after releasing their first brood into the brood pouch using imageJ software (https://imagej.nih.gov/ij/index.html).

The experimental design is not full factorial because of the number of genotypes and conditions tested in parallel. However, because genotypes were fixed across experimental conditions and generations, we were able to control for confounding factors e.g. genetic changes occurring from one generation to the next and genetic variation among experimental exposures, enabling WGP and TGP effects to be studied in isolation.

### Life history traits: genetic and plastic responses to photoperiod and temperature cues

We assessed evolutionary mechanisms (genetic changes, WGP, TGP, and their interactions) of response to early spring warming on life history traits using linear mixed models (LMMs) in the “lme4” package in R v.3.3.3^44^. We quantified the effects of Generation, Population, Treatment and their interactions on individual life history traits. Genotype was fit as a random effect nested within population. Prior to the analyses, all variables were tested for normality. Except for male proportion (a binomial variable), all life history trait measurements followed a Gaussian distribution. Because the populations separated in time originate from the same genetic pool and because genetic drift is negligible^30^, a significant population term indicates genetic differences among populations. Differences in mean trait values between photoperiods, after controlling for maternal effects, are the expression of WGP, an environmental effect. Differences in mean trait values between generations are the expression of TGP. If the effect of the treatment (photoperiod) differed significantly among populations (genetic effect), we would have evidence of a Pop (population) × Pht (photoperiod) interaction. Similarly, if the effect of the treatment (photoperiod) differed significantly among generations, we would have evidence of Gen (generation) × Pht (photoperiod) interaction. If population means varied by generation, we would have evidence of a Gen (generation) × Pop (population) interaction. We also measured the three-way interaction term (Gen × Pop × Pht). All evidence of interactions or main effects were assessed via Type II analysis of deviance tables using the Anova function in the “car” package (R v.3.3.3)^45^. We visualized the main effects of population (P1, P2 and P3), treatment (SP and LP) and generation (G1 and G2) plus their interaction terms on individual life history traits through reaction norms, which describe the pattern of phenotypic expression of each population across treatments and generations^46^.

We performed a principal component analysis (PCA) to quantify the principal modes of variation and covariation among life history traits within generation using the ‘prcomp’ function in the R “stats” package. The PCA plots were obtained using the “ggbiplot” (R v.3.3.3^44^). Prior to the PCA analysis, the life history traits variables were log transformed.

Mortality rates per population were calculated with a survival model fit via the “psm” function in the rms R package V.3.3.3^44^. A separate model was fitted to each treatment and generation, in which the day of mortality and the mortality event were combined as the dependent variables (e.g. censoring) and population was treated as fixed effect. All mortality curves were plotted using the “survplot” function form “rms” package in R v.3.3.3^44^.

### Candidate gene analysis: genetic responses to temperature and photoperiod cues

The genome of the 30 genotypes used in this study has been sequenced and will be published elsewhere. Here, we use the sequenced genome to identify SNP polymorphisms at 15 candidate genes previously associated with environmentally driven local adaptation, including male formation and the induction of sexual reproduction^40,41^ by mapping the sequences of the candidate genes against the reference genome of *D. magna* 2.4 (NBCI: LRGB00000000.1). The panel of SNPs at the 15 candidate genes is shown in Table S2. Methods used for mapping and variant calling are in Supporting Data 1.

To assess whether the candidate genes were under selection in the populations studied here, we used measures of genetic differentiation (F_ST_) and neutrality tests. Previous results identified a small yet significant proportion of neutral genetic divergence among the *D. magna* populations studied here (1%)^30^. Larger divergence among populations at the 15 candidate genes would identify genes under divergent selection, whereas a smaller divergence at the candidate loci would suggest balancing selection. In the former, different polymorphic sites at the same genes are found in different populations, resulting in higher divergence than at neutral loci. Conversely, balancing selection reduces population differentiation, because of moderate to intermediate frequencies of polymorphic sites^47^.

We quantified pairwise population differentiation as F_ST_, using MSA Analyser^48^, and the partitioning of genetic variance within and among populations (AMOVA) using Arlequin^49^. The two hierarchical levels used in the analysis are (i) among populations and (ii) within populations. Statistically significant values (P < 0.001) were calculated with permutation tests (10,000 permutations). Further, we performed neutrality tests on the candidate genes to quantify departure from neutrality using DnaSP^50^. Typically, these tests are affected by demography. In our study, the three populations are snapshots of the same genetic pool at different time points across five decades. Therefore, demographic confounding factors are negligible.

To assess whether the candidate genes were associated with life history traits showing responses to photoperiod, we performed a gene-trait association analysis between the candidate gene polymorphisms and life history traits measured in G1, using Mixed Linear Models in TASSEL^51^ with the following criteria: minor allele frequency (MAF) > 0.05 and Hardy-Weinberg Equilibrium (HWE) > 0.001, applying a false discovery rate of FDR = 0.05 to correct for multiple testing. We investigated significant association between gene polymorphisms at the 15 candidate genes and the life history traits in long and short photoperiod, separately. We also quantified association between the gene polymorphisms and the plastic change in life history traits as delta values between long and short photoperiod within G1.

## Results

### Life history traits: genetic and plastic responses to photoperiod and temperature cues

We investigated the impact of photoperiod and temperature cues on fitness-linked life history traits in *D. magna*. Short photoperiod at 20 °C mimicked early spring warming, whereas long photoperiod at 20 °C mimicked a typical spring environment, acting as reference.

We quantified the effects of Generation, Population, Treatment and their interactions on individual life history traits using an analysis of variance. The three-way interaction term (Generation × Population × Photoperiod) was significant for the proportion of male offspring (Table 1). The effect of photoperiod varied significantly between generations in four of the five life history traits (Table 1; Gen × Pht). The effect of photoperiod did not vary significantly by population, except for the proportion of male offspring (Table 1; Pop × Pht). A significant interaction term between generation and population was only observed for the proportion of male offspring (Table 1; Gen × Pop). We detected no difference among populations in trait means, except for fecundity (Table 1; Pop). All mean trait values differed significantly between generations (Table 1; Gen). The effect of photoperiod was significant on fecundity, size at maturity and time elapsing between broods (Table 1; Pht). Mortality was negligible in all experimental conditions (Table 1; Fig. S2).

**Table 1:** Analysis of variance.

	Fecundity			Size at maturity			Age at maturity			Male proportion			Average interval between broods			Mortality		
	Df	Chisq	p-value	Df	Chisq	p-value	Df	Chisq	p-value	Df	Chisq	p-value	Df	Chisq	p-value	Df	Chisq	p-value
Generation (Gen)	1	0.48	**<0.001**	1	23.09	**<0.001**	1	13.33	**0.001**	1	114.60	**<0.001**	1	19.15	**0.001**	1	2.04	0.15
Population (Pop)	2	10.52	**0.005**	2	0.11	0.94	2	0.21	0.90	2	3.89	0.14	2	0.96	0.62	2	5.35	0.07
Photoperiod (Pht)	1	31.89	**<0.001**	1	20.71	**<0.001**	1	0.47	0.49	1	1.31	0.25	1	41.23	**0.001**	1	2.30	0.13
Pop x Pht	2	1.47	0.48	2	4.21	0.12	2	0.03	0.98	2	39.55	**<0.001**	2	2.41	0.30	2	5.27	0.07
Pop x Gen	2	2.86	0.24	2	0.83	0.66	2	4.32	0.11	2	18.82	**<0.001**	2	1.39	0.50	2	4.78	0.09
Pht x Gen	1	16.64	**<0.001**	1	12.25	**<0.001**	1	13.55	**0.001**	1	4.80	**0.028**	1	2.02	0.15	1	2.04	0.15
Pop x Pht x Gen	2	0.38	0.83	2	0.13	0.94	2	0.69	0.71	2	26.70	**<0.001**	2	2.00	0.37	2	4.75	0.09

Univariate ANOVAs per single life history traits (fecundity over the life span of the genotypes, size at maturity (mm), age at maturity (days), proportion of males offspring over the life span of the genotypes, the time elapsed between broods averaged over eight broods (Av. time interval between broods, days) and mortality are shown. Generation (Gen), Population (Pop), Photoperiod (PhT), and their interaction terms are shown. Significant *P-values* are in bold.

**Figure 2:**
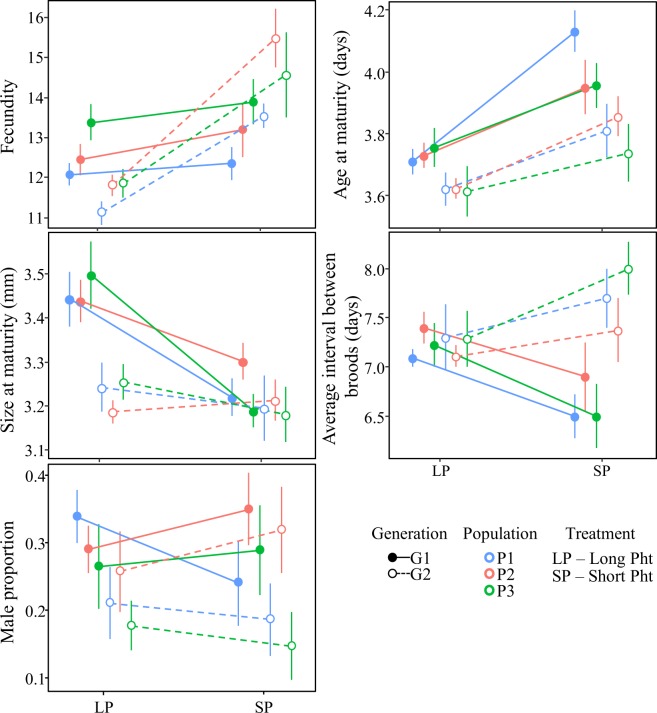
Life history traits response to changes in photoperiod. Population reaction norms based on population means (N = 9–11) and SE measured in long photoperiod (LP) and short photoperiod (SP) for generation 1 (G1; continuous lines) and generation 2 (G2; dotted lines). The life history traits measured are: fecundity, age at maturity (days), size at maturity (mm); proportion of male offspring, and average interval between broods (days). The populations are colour coded as in Fig. 1: P1 – blue; P2 – red; P3 – green. Statistical analyses supporting the reaction norms are in Table 1.

Short photoperiod caused a decrease in size and in the time elapsing between parthenogenetic broods in the first experimental generation (Fig. 2; G1). In the second generation (G2), short photoperiod induced increase in fecundity, smaller size at maturation, and longer time elapsing between parthenogenetic broods (Fig. 2; G2). Male proportion varied with generation and population (Fig. S1), showing both genetic and plastic responses (Fig. 2).

**Figure 3:**
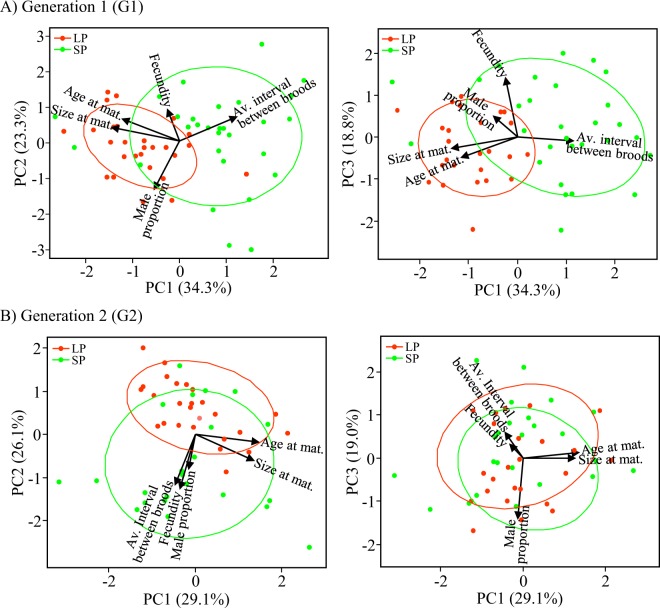
Principal component analysis. PCA plots showing phenotypic plasticity at five fitness-linked life history traits measured in (**A**) generation 1 (G1) and (**B**) generation 2 (G2) in long photoperiod (LP, 14:10 h light: dark regime; orange) and short photoperiod (SP, 10:14 h light: dark regime; green). Patterns are given for PC1 and PC2 and PC1 and PC3. The life history traits are the same as in Table 1: age at maturity, size at maturity, fecundity, male proportion and average interval between broods.

We observed a clear difference in the trait trade-offs between generations and experimental conditions (Fig. 3). In G1, PC1 was positively correlated with average time elapsing between broods (PC1: 52%) and negatively correlated with age and size at maturity (PC1_age_: −53%; PC1_size_: −62%). PC2 was positively correlated with fecundity (PC2: 48%) and negatively with the proportion of male offspring (PC2: −71%). PC3 was positively correlated with fecundity (PC3: 87%) and negatively with size and age at maturity (PC3_size_: −16%; PC3_age_: −30%) (Fig. 3A). In G2, PC1 was positively correlated with age and size at maturity (PC1_age_: 70%; PC1_size_: 65%), whereas PC2 negatively correlated with fecundity, the proportion of male offspring and the time elapsing between broods (PC2_fecundity_: −62%; PC2_male_: −41%; PC2_av. interval_: −58%). In this generation, PC3 was negatively correlated with the proportion of male offspring (PC3: 89%) and positively correlated with fecundity and average time elapsing between broods (PC3_fecundity_: 21%; PC3_av. interval_: 39%) (Fig. 3B).

### Candidate gene analysis: genetic responses to temperature and photoperiod cues

The genetic divergence among populations was higher at neutral loci than at the candidate genes in presence of negligible drift (Table 2, Table S3). The neutrality test on the candidate genes identified nine out of fifteen genes as putatively under selection; at least two independent tests supported departure from neutrality in these genes (Table 3). The candidate genes showed significant association with fecundity and male offspring proportion both in short and long photoperiod (Table S4; SP, LP). Fecundity showed significant association with the histone deacetylase complex subunit SAP18 (Table S4). The proportion of male offspring showed significant association with Amino-oxidase, SAP18, Phosphotyrosine (PTB), and Aldo/keto reductase (Table S4). In addition, the candidate genes showed significant association with plastic changes in all traits (Table S4; Delta). Plastic changes in age at maturity were significantly associated with the rhodopsin g-protein coupled receptor; plastic changes in fecundity were significantly associated with the Histone deacetylase complex subunit SAP18; variation in male offspring proportion was significantly associated with Amino-oxidase, SAP18, Phosphotyrosine (PTB), and Aldo/keto reductase; plastic change in size at maturity was significantly associated with Pyridine nucleotide-disulphide oxidoreductase, PTB, SAP18, and an RNA binding protein (Table S3).

**Table 2:** Analysis of molecular variance.

	Among populations	Within populations
Neutral µsat	1.08*	98.92*
Candidate genes	0	100*

AMOVA analysis showing the partitioning of genetic variance within and among populations at neutral microsatellite loci and at the 15 candidate genes. The data for the microsatellite loci are from^30^. The two hierarchical levels used in the analysis are (i) among populations and (ii) within populations. Statistically significant values (*P < 0.001) are based on permutation tests (10,000 permutations).

**Table 3:** Neutrality tests.

GeneID	ScaffoldID	Start	End	S	Pi	θ	Gene function	Ref	Tajima’s D	Fu&Li’s D	Fu&Li’s F
Dapma7bEVm001004t1	scaffold00027	2877	6078	23	0.36	0.21	Serine arginine-rich splicing factor 7	Reisser *et al*.^40^	2.13*	1.76**	2.25**
Dapma7bEVm005301t1	scaffold00848	96321	97283	5	0.29	0.21	Aldo-keto reductase family 1, member C4	Reisser *et al*.^40^	0.8	1.08	1.16
Dapma7bEVm002245t1	scaffold02003	35289	35935	2	0.3	0.21	Poly-U-binding splicing factor Half Pint	Reisser *et al*.^40^	0.71	0.73	0.84
Dapma7bEVm015923t3	scaffold02003	213333	214454	48	0.45	0.22	Cytochrome P450 314 family	Reisser *et al*.^40^	3.62***	2.03**	3.14**
NA	scaffold02569	3227	4315	7	0.39	0.21	Zinc transporter zip11	Reisser *et al*.^40^	2.14*	1.23	1.79*
Dapma7bEVm006598t1	scaffold02569	9179	10907	12	0.3	0.21	Zinc transporter zip9	Reisser *et al*.^40^	1.19	1.48*	1.63
Dapma7bEVm008171t1	scaffold02569	35151	44725	91	0.42	0.22	SOX-9-like transcription factor	Reisser *et al*.^40^	3.07**	2.18**	3.02**
Dapma7bEVm002217t1	scaffold02569	218892	220701	4	0.36	0.21	DnaJ homolog dnaj-5	Reisser *et al*.^40^	1.44	0.99	1.32
Dapma7bEVm028519t1/Dapma7bEVm010615t1	scaffold02569	334258	337000	79	0.21	0.22	Broad-complex	Reisser *et al*.^40^	−0.19	2.15**	1.5
Dapma7bEVm004407t1	scaffold02569	340469	342584	20	0.21	0.21	Transformer2	Reisser *et al*.^40^	−0.01	1.70**	1.3
Dapma7bEVm000710t1	scaffold02569	76814	79370	19	0.42	0.21	Protein SPT2 homolog	Reisser *et al*.^40^	2.92**	2.92**	2.52**
Dapma7bEVm007919t1	scaffold02569	228772	229714	7	0.45	0.21	Histone deacetylase complex subunit sap18	Reisser *et al*.^40^	2.83**	1.23	2.06**
Dapma7bEVm005463t1	scaffold02723	1124	6033	37	0.31	0.22	Epidermal growth factor receptor kinase	Reisser *et al*.^40^	1.33	1.95**	2.05**
Dapma7bEVm001751t1	scaffold03156	4200	8559	40	0.27	0.21	Lysine-specific histone demethylase 1A	Reisser *et al*.^40^	0.89	1.96**	1.87*
Dapma7bEVm015675t1	scaffold01036	708969	713276	64	0.37	0.22	Rhodopsin	Roulin *et al*.^41^	2.32*	2.10**	2.61**

Statistics testing for departure from neutrality of 15 candidate genes previously associated with environmental driven local adaptation and partial sex determination. For each candidate gene (GeneID), the scaffold location (ScaffoldID), start and end position of the gene, nucleotide diversity (S), theta per site (θ), gene function and bibliographic reference are shown. For each gene, the result of Tajima’s D^77^, Fu&Li D and F tests^78^ are shown. Significant values, calculated with a FDR = 0.05, are marked with an asterisk (*).

## Discussion

Investigating both plasticity and genetic adaptation across generations enables to establish a link between evolutionary potential (either additive genetic variation or genotype by environment interactions) and TGP^52–54^. We used transgenerational common garden experiments on resurrected *D. magna* populations separated in time to study the mechanisms of response – genetic, plastic (WGP, TGP) or a combination thereof - to earlier spring warming. Resurrection ecology provided us with the unique advantage of following evolution *in situ* across decades by studying genetic differences among populations that evolved from an initial genetic pool in response to environmental selection^55,56^. The use of genetically distinct clonal lineages across generations enabled us to perform the concurrent analysis of plastic and genetic mechanisms of adaptation to environmental change within and across generations minimizing confounding factors.

We observed significant difference in the plastic response to spring warming between generations, made evident from a significant generation term and a significant interaction term Generation × Photoperiod in the ANOVA. Trade-offs among life history traits clearly differed between generations. In G1 the time elapsing between parthenogenetic reproductions showed trade-offs with age and size at maturity, and fecundity showed trade-offs with the proportion of male offspring. In G2, size and age at maturation showed trade-offs with all other life history traits; moreover, the investment in male offspring came at a cost of fecundity and time elapsing between parthenogenetic reproductions.

Theory predicts that highly plastic traits show strong maternal effect variance and little to no genetic variance, because highly plastic traits can be influenced by the environment, including parental environment, and because additive genetic variance may be masked by high environmental variation^57^. For example, elytron length in seed beetles, a highly plastic trait, is strongly influenced by parental effects and has no detectable genetic variance^58^; body and offspring size in stickleback is strongly influenced by the parental environment but does not show high genetic variance^59^. Our results suggest a positive maternal effect on earlier spring warming when the offspring environment perfectly matches the maternal one. This effect is evident from fecundity, which is higher in G2 than G1, and from size at maturity that does not change significantly in G2 but significantly decreases in G1 in short photoperiod. However, a full factorial design measuring the anticipatory maternal effect in matching and not matching environments is needed to confirm positive maternal effect in matching environments^13^. In addition, the photoperiod cue experienced by the second generation during fertilization may have influenced the phenotypic response of the offspring generation, result of developmental plasticity. The timing at which the second generation directly experience the parental environment has been shown to influence offspring response in matching environments^60,61^. In particular, exposure during fertilization and early juvenile development may strongly affect offspring response to parental environments^2^. Follow up experiments, in which the parental generation is exposed for different lengths of the life cycle to early spring warming, is needed to exclude whether exposure during fertilization influenced offspring response to early spring warming.

We observe a strong role of WGP in response to earlier spring warming, made evident from significant photoperiod effect on the majority of life history traits. The effect of photoperiod resulted in smaller size at maturation, delayed maturation and a decrease in the time lapse between parthenogenetic reproductions in G1, whereas it resulted in higher fecundity, and an increase in the time between parthenogenetic reproductions in G2.

We observe genetic responses to early spring warming quantified as genetic differences among populations in fecundity. Moreover, we observe a lower than neutral genetic divergence at the candidate loci, which were significantly departing from neutrality expectations. The lake from which the *Daphnia* populations were sampled has a documented increase in average ambient temperature and recurrence of heat waves over time^36^. Previous studies on these populations showed evidence for temporal evolution of the critical thermal maximum (CT_max_), the upper temperature at which animals lose motor function^62^, in presence of warming as a single stress^63^. Here, we observe significant evolutionary differences among populations to early spring warming cues (G1). These evolutionary differences indicate that the most recent population evolved to be less fecund than the historical populations. This may be the result of maladaptation or of evolutionary responses to multiple stressors. It is possible that multiple stressors documented in Lake Ring (e.g changes in water transparency and lake chemistry^39^) have influenced adaptive responses of the population over time^64^. Indeed, in a previous study on the populations of Lake Ring, higher CT_max_ in the most recent population was observed in presence of warming alone, whereas it was no longer observed in presence of multiple stressors^63^.

In the presence of stable neutral genetic diversity over time^30^, the candidate genes show a departure from neutrality and a lower divergence among populations than neutral markers. These patterns are expected when balancing selection affects the frequency of gene polymorphism across populations^47^. The candidate genes putatively under selection in the studied populations have been previously linked to functions in crustaceans and insects relevant in the context of dormancy, sexual reproduction, male formation and general stress responses: Rhodopsin has been associated with dormancy in *Daphnia*^41^; SAP18 has been associated with embryonic development and environmental stress response in insects^65,66^; PTB (epidermal growth factor receptor kinase) has been linked to sexual differentiation in insects and crustaceans^67,68^; MF, a catalyser for the methyl farnesoate and a putative juvenile hormone in daphniids, and AKR (aldo-keto reductase) have been linked to the propensity to form male offspring^69,70^. In the MF pathway, the AKR family catalyses the conversion of farnesal to farnesol, and mutations in the AKR gene have been shown to reduce MF production in favour of the juvenile hormone, resulting in higher male production^70,71^. The function of these genes and their significant association with life history traits in the population studied here suggest their potential role in the response to photoperiod and temperature cues. However, it is possible that other environmental stressors, which have been documented in Lake Ring, may have contributed to the observed response at the candidate genes and/or that additional genes not investigated here are contributing to modulate the life history traits analysed. Indeed, all traits showed significant association with multiple candidate genes suggesting epistasis among genes underpinning the life history traits. Furthermore, single genes showed association with multiple traits, suggesting extensive pleiotropy. It is expected that multiple genes regulate complex fitness-linked life history traits. In a follow up experiment, we generated further evidence supporting this expectation (Supporting Data 1). We used three genotypes that showed divergent patterns in male offspring proportion between photoperiods (Fig. S1) and a reference genotype, which never produces male broods under all tested experimental conditions to date (P-IT, Institute of Ecosystem study, CNR Verbania, Italy)^72^. We focused on the propensity to form males because some of the candidate genes studied here have been previously associated with this trait^40,69,70^. Fixed polymorphisms among the strains with divergent propensity to form male offspring would suggest that the candidate genes are underpinning this trait. Lack of evidence for fixed polymorphisms at the candidate genes would suggest that other genes underpin male offspring formation. We found that the divergent patterns in propensity to form male offspring in the four genotypes did not correspond to fixed polymorphisms at the 15 candidate genes, confirming pleiotropy and the need to investigate genome-wide patterns to identify the genes underpinning complex fitness traits (Supporting Data 1).

Overall, our results point to an adaptive potential for a keystone zooplankter *Daphnia* to evolve in response to early spring warming mediated by a complex interaction between plastic and genetic mechanisms. The results also suggest a positive maternal effect in presence of matching environments between parental and offspring generations. A positive maternal effect at a life history trait indicates accelerated rates of microevolution that can facilitate adaptation^73^. However, a full factorial design is needed to assess whether non-matching environments provide different responses.

We identified extensive pleiotropy in candidate genes underpinning life history traits responsive to early spring warming. Pleiotropy is commonly observed in complex environments with multifarious selection pressures acting on multiple aspects of the phenotypes, resulting in trade-offs among competing functions^74–76^. This finding supports previous experimental results showing that populations in complex environments can overcome fitness cost exhibiting synergistic pleiotropy^74^.

## Supplementary information

Supporting Tables and Figures

Table S2

Table S4

SUPPORTING DATA 1
