# Supplementary material for: Transgenerational response to early spring warming in Daphnia
Source: Sci Rep. 2019 Mar 14;9:4449. doi: 10.1038/s41598-019-40946-3 (PMC6418131; doi:10.1038/s41598-019-40946-3)
Supplement: Supplementary file 1 — Supporting Tables and Figures [file 41598_2019_40946_MOESM1_ESM.docx]

**Transgenerational response to early spring warming in *Daphnia***

**Running title: Early spring warming in *Daphnia***

Kenji Toyota^1,2,3^, Maria Cuenca Cambronero^1,*#^, Vignesh Dhandapani^1*^, Antonio Suppa ^1,4^, Valeria Rossi^4^, John K. Colbourne^1^, Luisa Orsini^1^

^1^ Environmental Genomics Group, School of Biosciences, University of Birmingham, Birmingham B15 2TT, UK

^2^ Department of Biological Science, Faculty of Science, Kanagawa University, Hiratsuka, Kanagawa, 259-1293, Japan.

^3^ Department of Biological Science and Technology, Tokyo University of Science, Katsushika, Tokyo, Japan.

^4^ Department of Chemistry, Life Sciences and Environmental Sustainability University of Parma, Department of Life Sciences, Viale Usberti, 11/A Parma, Italy

^#^ Current address: Aquatic Ecology Department, EAWAG, Kastanienbaum, Switzerland

Corresponding author:

Dr Luisa Orsini

Environmental Genomics Group, School of Biosciences, University of Birmingham

Birmingham, B15 2TT, United Kingdom

T: +44 (0)121 4145894 F: +44 (0)121 414 5925

Email: [l.orsini@bham.ac.uk](mailto:l.orsini@bham.ac.uk)

*these authors contributed equally

**Table S1. List of genotypes used in this study**

List of *Daphnia magna* genotypes used in the common garden experiments to identify life history trait response to photoperiod changes. All genotypes were resurrected from Lake Ring, Denmark. Genotype ID and population ID are as in Cambronero *et al.* (2017). P1: population resurrected from layers of sediment dated >1999; P2: population resurrected from layers of sediment dated 1975-1985; P3: population resurrected from layers of sediment dated 1960-1970.

| **GenotypeID** | **PopulationID** |
| --- | --- |
| LRV 0_1 | P1 |
| LRV 0_2 | P1 |
| LRV 0_4 | P1 |
| LRV 1_2 | P1 |
| LRV 2_1 | P1 |
| LRV 2.5_9 | P1 |
| LRV 2.5_11 | P1 |
| LRV 3_6 | P1 |
| LRV 3.5_1 | P1 |
| LRV 3.5-2 | P1 |
| LRV 3.5_15 | P1 |
| LRV 6_2 | P2 |
| LRV 6_3 | P2 |
| LRV 7_3 | P2 |
| LRV 7_5 | P2 |
| LRV 7.5_4 | P2 |
| LRV 8.5_3 | P2 |
| LRV 9_6 | P2 |
| LRV 9_2 | P2 |
| LRV 9.5_1 | P2 |
| LRV 9.5_3 | P2 |
| LRV 12_3 | P3 |
| LRV 12_4 | P3 |
| LRV 12.5_1 | P3 |
| LRV 13_1 | P3 |
| LRV 13_2 | P3 |
| LRV 13_3 | P3 |
| LRV 13.5_1 | P3 |
| LRV 14.5_1 | P3 |
| LRV 15.5_1 | P3 |

**Table S2. SNP variants at candidate genes**

Single nucleotide polymorphisms are show at 15 candidate genes previously associated with environmental driven local adaptation in *D. magna*. *D. magna* geneID; reference scaffold on the *D. magna* reference genome v2.4; SNP position on scaffold; SNP variant on the *D. magna* reference genome; and allelic variants across the 30 genotypes are shown. Genotype’s names are as in Table S1. SNP polymorphisms are shown in IUPAC codes: M: A/C; R: A/G; Y: C/T; K: G/T; S: G/C; W: A/T.

See excel file Toyota *et al.* Table S2.

**Table S3. Population divergence**

Pairwise genetic differentiation among populations calculated as F_ST_ at neutral loci (A) and at the 15 candidate genes previously associated with environmental driven local adaptation in *D. magna* (B). Values for neutral genetic markers are from Orsini *et al*., 2016. The candidate loci are listed in Table 1. Asterisks (**) indicate significant pairwise differentiation.

| A |  |  |  |  | B |  |  |
| --- | --- | --- | --- | --- | --- | --- | --- |
|  | P1 | P2 | P3 |  | P1 | P2 | P3 |
| P1 | - | ** | ** |  | - |  |  |
| P2 | 0.011 | - | ** |  | 0.014 | - |  |
| P3 | 0.016 | 0.016 | - |  | 0.014 | 0.009 | - |

**Table S4. Gene-trait association at candidate genes**

SNPs at 15 candidate loci showing significant associations with life history traits (fecundity, size, age at maturity, and proportion of male offspring measured across eight broods) measured in the common garden experiment for G1 under short photoperiod (SP), long photoperiod (LP), and plastic variation between long and short photoperiod (Delta Pval). For each SNP, the ScaffoldID, the geneID on the *D. magna* reference genome v2.4 and gene function as identified in blast searches are shown. P-values > 0.05 are shown.

See excel file Toyota *et al.* Table S4.

**Figure S1**. **Males produced in different photoperiods**

The percentage of male offspring in the 30 genotypes of *D. magna* used in the common garden experiment, separated per population (P1, P2, P3) and generation (G1, G2). Short photoperiod (SP) is shown in green and long photoperiod (LP) is shown in orange. Genotype names are as in Table S1. Black boxes are for genotypes used in the validation experiment described in Supplementary Data1.


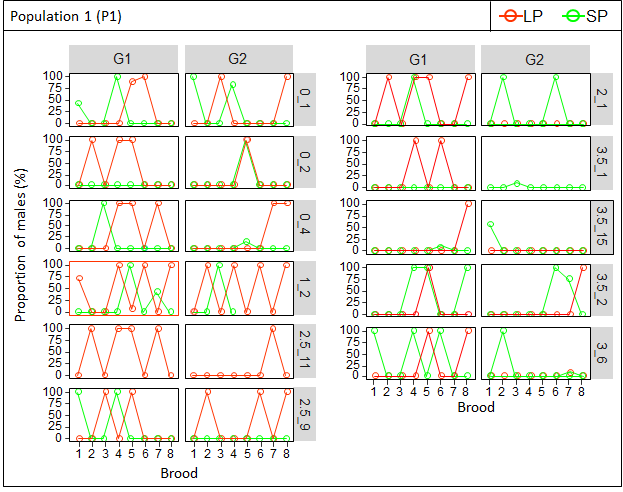


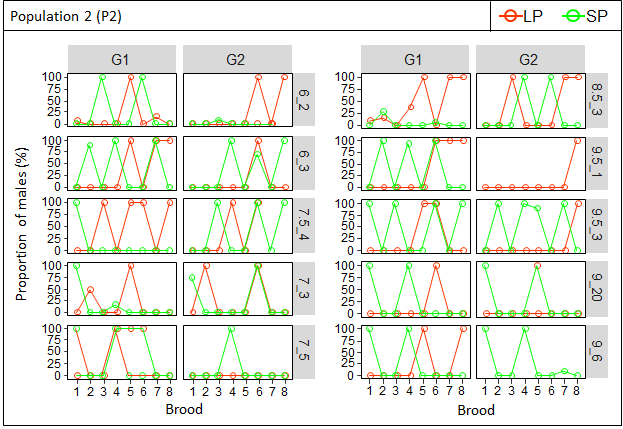


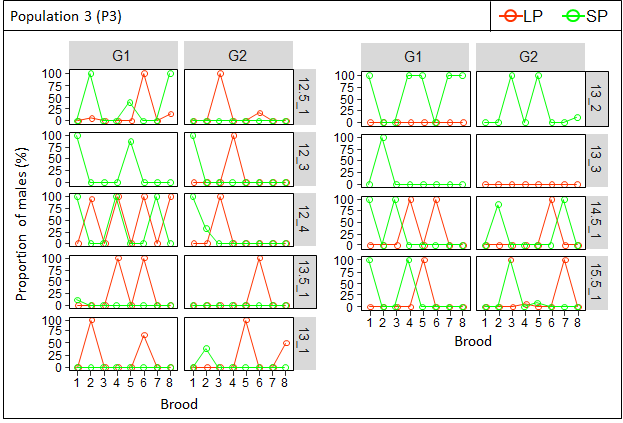


**Figure S2. Mortality curves CGE1**

Mortality plots per population in generation 1 (G1) and generation 2 (G2), calculated with a survival model fit via the “psm” function in the “rms” R package V.3.3. A separate model was fitted to each treatment and generation, in which the day of mortality and the mortality event itself were treated as dependent variables, whereas population was treated as fixed effect. Populations are color-coded as in Figure 1.


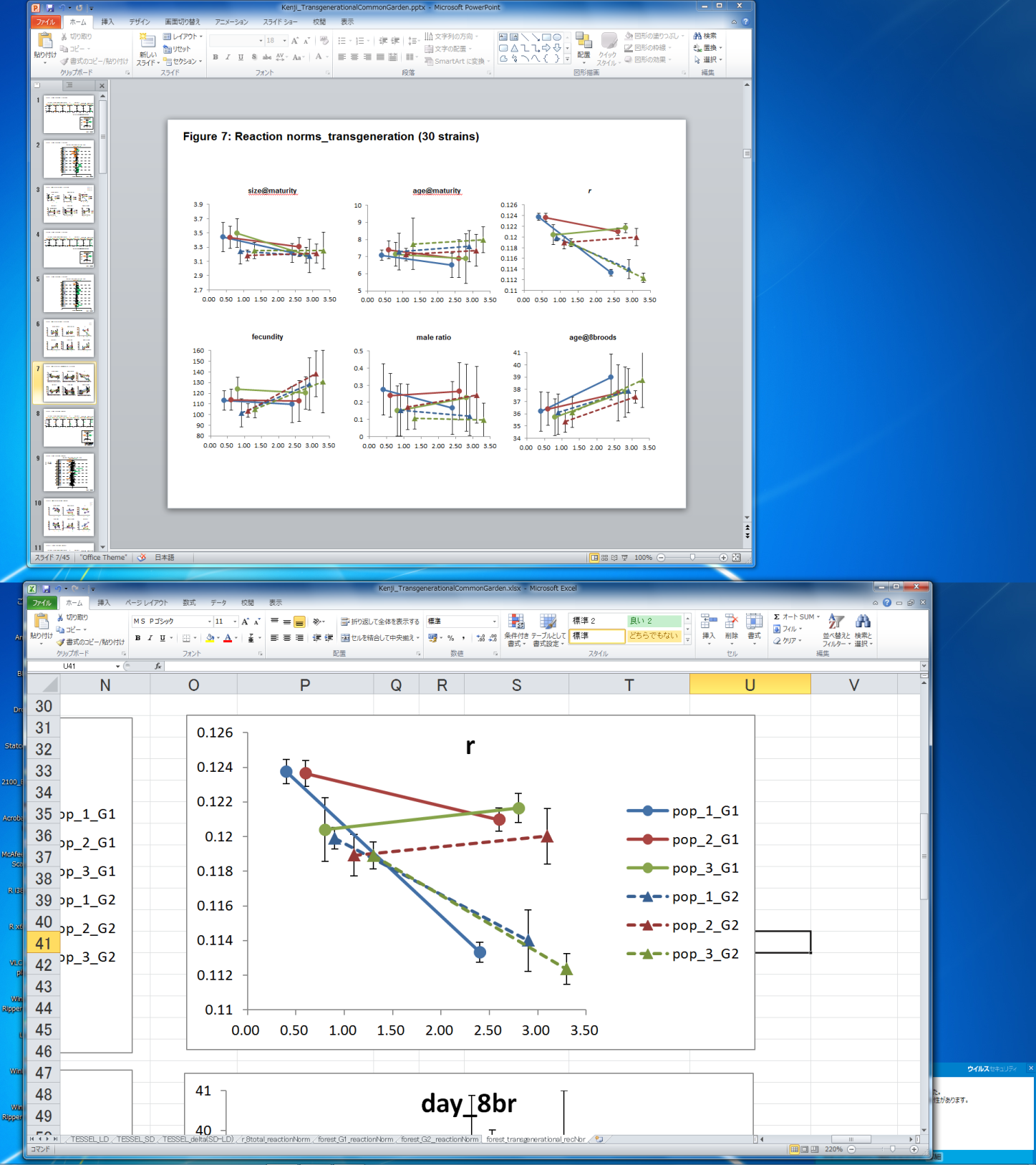


P1

P2

P3

0

40

0.0

0.2

0.4

0.6

0.8

1.0

Generation 1 (G1)

Generation 2 (G2)

0

40

0.0

0.2

0.4

0.6

0.8

1.0

Short photoperiod (SP)

Days

0

40

0.0

0.2

0.4

0.6

0.8

1.0

Days

0

40

0.0

0.2

0.4

0.6

0.8

1.0

Long photoperiod (LP)
