# Supplementary material for: Transgenerational response to early spring warming in Daphnia
Source: Sci Rep. 2019 Mar 14;9:4449. doi: 10.1038/s41598-019-40946-3 (PMC6418131; doi:10.1038/s41598-019-40946-3)

**Transgenerational response to early spring warming in *Daphnia***

**Running title: Early spring warming in *Daphnia***

Kenji Toyota^1,2,3^, Maria Cuenca Cambronero^1,*#^, Vignesh Dhandapani^1*^, Antonio Suppa ^1,4^, Valeria Rossi^4^, John K. Colbourne^1^, Luisa Orsini^1^

^1^ Environmental Genomics Group, School of Biosciences, University of Birmingham, Birmingham B15 2TT, UK

^2^ Department of Biological Science, Faculty of Science, Kanagawa University, Hiratsuka, Kanagawa, 259-1293, Japan.

^3^ Department of Biological Science and Technology, Tokyo University of Science, Katsushika, Tokyo, Japan.

^4^ Department of Chemistry, Life Sciences and Environmental Sustainability University of Parma, Department of Life Sciences, Viale Usberti, 11/A Parma, Italy

^#^ Current address: Aquatic Ecology Department, EAWAG, Kastanienbaum, Switzerland

Corresponding author:

Dr Luisa Orsini

Environmental Genomics Group, School of Biosciences, University of Birmingham

Birmingham, B15 2TT, United Kingdom

T: +44 (0)121 4145894 F: +44 (0)121 414 5925

Email: [l.orsini@bham.ac.uk](mailto:l.orsini@bham.ac.uk)

*these authors contributed equally

**SUPPORTING DATA 1: additional methods and validation experiment**

**Lake Ring paleolimnological profile**

The *D. magna* populations separated in time used in this study were previously ‘resurrected’ from a biological archive from Lake Ring, a well characterized shallow mixed lake (without thermocline stratification, 22.5 hectares) in Jutland, Denmark (55°57’51.83’’ N, 9°35’46.87’’ E) ^1^. According to historical records, sewage inflow from a nearby town, increased nutrient level in the lake starting from the 1950s and resulting in eutrophication ^2^. The sewage inflow was diverted in the 1960s. At this time agricultural land use intensified, and the lake suffered from pesticides and herbicides run off ^2^. The lake partially recovered from hyper-eutrophication in modern times (>1999s) but, as every lake in Europe, it experienced an increase in average ambient temperature (~1°C) ^3^. Data on temperature were collected over the past century by the Danish Meteorological Institute at a weather station located 80 km from Lake Ring (<http://www.dmi.dk/laer-om/generelt/dmi-publikationer/2013/>). Because air and water surface temperature have a positive correlation for shallow streams and lakes ^4,5^, we used the data from the weather station as estimate of the lake water temperature. In addition to temperature, other water properties were recorded: water transparency, total phosphorous and total nitrogen for the years 1971-1999. Moreover, records of pesticides historically sold in Denmark were available from the Danish national archives for the period 1955-2010. A detailed description of the paleolimnological profile of Lake Ring is in Cambronero *etal.* ^1^.

In 2004, Lake Ring was sampled using a piston corer of 6 cm internal diameter as described in ^6^. Sediment was sliced in layer of 0.5 cm and stored in dark and cold condition (4 °C). In 2015, the sedimentary archive was subjected to radiometric chronology ^7^, providing a reliable dating of each layer of sediment to the 1900. Threfore, the age of the three populations of *D.magna* is known.

Previously, the sedimentary archive of Lake Ring, was inspected for dormant *D. magna* embryos; from the archive, 262 dormant embryos were successfully hatched, following established protocols ^6^. Each hatchling is genetically distinct (unique genotype) as it is the result of sexual recombination. Clonal lineages established from the 262 genotypes are currently maintained in standard laboratory conditions (10 °C, 16:8 light: dark regime and 0.4 mg Carbon/L of *Chlorella vulgaris* bi-weekly). Of the 262 dormant embryos successfully hatched, we used 10 distinct genotypes per population in the present study. Each population correspond to a lake phases– sewage (1960-1970) referred to as the eutrophication phase (EP), pesticide (1975-1985) referred to as the pesticide phase (PP) and recovery (>1999) referred to as the clear water phase (CWP). In the current study, population 1 (P1), population 2 (P2) and population 3 (P3), are from the CWP, PP and EP phases, respectively.

The sample size per population was chosen based on previous results showing that 10 genotypes are representative of the local genetic diversity of *D. magna* populations ^8^. The resurrected genotypes are an unbiased representation of the local population genetic diversity as hatching success fluctuates along the sedimentary archive but did not systematically decrease with the age of the sediment ^6^. Previous results on the genetic composition of *D. magna* in Lake Ring showed that genetic drift and selection did not have a detectable impact on the neutral genetic diversity over time, measured both on the hatched and unhatched populations of *D. magna* throughout the sedimentary archive ^8^.

**SNP variant calling at the candidate genes**

The genome of the 30 genotypes of *D. magna* used in the experiments was sequenced (data to be published elsewhere). Here, we describe the main steps that led to the identification of SNP variants at the 15 candidate genes used in this study and listed in Table 3.

The genome sequence data were subjected to quality check by mapping raw reads onto the draft genome of *D. magna* v2.4 (Accession: LRGB00000000). The reads were then aligned and assembled in individual genomes; SNP variants were called on the assembled genomes using the pipeline depicted in Figure SD1. Read sequences basepair quality was assessed with Fastqc tools (<https://www.bioinformatics.babraham.ac.uk/projects/fastqc/>). Trimmomatic ver.0.33 was applied for adapter trimming and to remove low quality sequences ^9^. Paired-end reads with Q>30 and read length of 50bp were retained and mapped against the reference genome of *D. magna* using the BWA-mem algorithm ^10^ (Fig. SD1). Samtools were used for format conversion, sorting, indexing and merging of mapping files from multiple runs of the same genotype ^11^. Picard tools were used to mark and remove duplicated reads and realign the trimmed dataset with GATK ^12^. Allelic variants and indels were called via bcftools (https://samtools.github.io/bcftools/) after applying the samtools mpileup command (samtools ver. 0.1.19, 45). Finally, SNP and indel variants were filtered with vcftools v0.1.14. Filtering criteria were as follows: minimum read depth (DP) > 10; SNP calls Quality (Phred score) for each sample Q> 30; minor allele frequency (MAF) > 5%; minimum allele count > 5; maximum missing values 50%; Avg. genotype quality > 50.

**Validation of the genetic basis of male formation in *D. magna***

Here, we describe a follow up experiment which was designed to provide supporting evidence for pleiotropy in *D. magna*.

The 30 genotypes used in the common garden experiment, showed different propensity to form male offspring across conditions and generations (Fig. S1). Among the 30 genotypes, three showed a consistent pattern between generations and photoperiods: LRV3.5_15 produced only female broods in both photoperiods; LRV13_5.1 produced male broods in long photoperiod; and LRV13_2 produced male broods in short photoperiod. In an effort to identify fixed gene polymorphisms in the 15 candidate genes putatively associated with the propensity to form male offspring, we performed a follow up common garden experiment. In this experiment, male offspring formation was quantified in the three genotypes from Lake Ring and in a reference genotype (P-IT, Institute of Ecosystem study, CNR Verbania, Italy), which never produces male broods under all tested experimental conditions to date ^13^. We focused on the propensity to form male offspring because some of the candidate genes studied here have been previously associated with this trait ^14,15,16^. Fixed polymorphisms among the strains with divergent propensity to form male offspring would suggest that the candidate genes are underpinning this trait. Lack of evidence for fixed polymorphisms at the candidate genes would suggest that genes other than the ones studied here underpin male offspring formation.

We performed the common garden experiment using six clonal replicates. This experiment aimed at assessing the propensity to form male offspring in the three strains from Lake Ring and the reference strain in the same experimental set up. We then inspected SNP polymorphisms at the candidate genes in the four genotypes to identify genetic patterns of divergence between strains showing different propensity to form male offspring.

Prior to the experiment, clonal lines established from the four genotypes were maintained for two generations in common garden conditions (20 °C, 12:12 h light: dark regime; fed *ad libitum* with 0.8 mg Carbon/L of *Chlorella vulgaris* CCAP strain no. 211/11B) to reduce potential inference from maternal and grandmaternal effects. Individual juveniles of 24 h from the second or following broods of the second generation in common garden conditions were randomly assigned to long (LP; 14:10 h light: dark regime) and short (SP; 10:14 h light: dark regime) photoperiod. The proportion to form male offspring was measures across 8 broods.

To be able to study polymorphisms across the four strains, we sequenced the genome of the reference laboratory strain P-IT (NCBI SRA database: SRX4042391) which we mined for polymorphisms at the 15 candidate genes. To generate the panel of polymorphisms we extracted genomic DNA (gDNA) using Agencourt DNA Advance (Beckman Coulter - A48706) with minor modifications and quantified using a ND-8000 Nanodrop (Thermo Fisher Scientific - ND-8000-GL). Up to 1µg of gDNA per genotype was sheared using a Bioruptor^®^ Pico ultrasonicator with integrated cooling module (Diagenode - B01060010), following cooling on ice for 10minutes. Sheared gDNA was assayed on a 2200 TapeStation (Agilent) with High Sensitivity DNA Screentapes to determine the distribution of sheared fragments. The sheared gDNA was then prepared into Illumina compatible DNA Sequencing 250bp paired-end libraries using KAPA HyperPrep Kit (Roche - KK8504), without amplification step. Following library construction, libraries were assayed and quantified on a 2200 TapeStation (Agilent) with High Sensitivity DNA Screentapes. Libraries were normalized to an average concentration of 2 nM prior to pooling and sequenced on an Illumina4000 sequencer. Allelic variants and indels were identified by mapping against the low-recombining genomic region containing the 15 candidate genes on the reference *D. magna* genome (v 2.4) following the steps shown in Figure SD1.

The follow up common garden experiment confirmed that LRV3.5_15 produced only female broods in both photoperiods; LRV13_5.1 produced male broods in long photoperiod; LRV13_2 produced male broods in short photoperiod; and P-IT never produced males (Fig. SD2). These patterns did not correspond to fixed polymorphisms at the 15 candidate genes (Table SD1).

**Supporting data references**

1 Cambronero, C. M. *et al.* Predictability of the impact of multiple stressors on the keystone species Daphnia *Sci Rep-Uk* **8**, 17572 (2018).

2 Michels, H. *Micro-evolutionary response of Daphnia magna to changes in biotic stress associated with habitat degradation and restoration of a shallow lake* Biology thesis, University of Leuven, (2007).

3 IPCC. Summary for policymakers 1-32 (Cambridge, United Kingdom and New York, NY, USA, 2014).

4 Preudhomme, E. B. & Stefan, H. G. Relationship between water temperatures and air temperatures for central U.S. streams. (University of Minnesota, St. Anthony Falls hydraulic Laboratory, Duluth, Minnesota, 1992).

5 Livingstone, D. M. & Lotter, A. F. The relationship between air and water temperatures in lakes of the Swiss Plateau: a case study with palæolimnological implications. *Journal of Paleolimnology* **19**, 181-198 (1998).

6 Cambronero Cuenca, M. & Orsini, L. Resurrection of dormant Daphnia magna: protocol and applications. *JoVE* **131**, e56637,, doi:doi:10.3791/56637 (2018).

7 Appleby, P. G. *Chronostratigraphic techniques in recent sediments*. Vol. 1 (Kluwer Academic Publisher, 2001).

8 Orsini, L. *et al.* Temporal genetic stability in natural populations of the waterflea Daphnia magna in response to strong selection pressure. *Molecular Ecology* **25**, 6024-6038, doi:10.1111/mec.13907 (2016).

9 Bolger, A. M., Lohse, M. & Usadel, B. Trimmomatic: a flexible trimmer for Illumina sequence data. *Bioinformatics* **30**, 2114-2120, doi:10.1093/bioinformatics/btu170 (2014).

10 Li, H. & Durbin, R. Fast and accurate long-read alignment with Burrows-Wheeler transform. *Bioinformatics* **26**, 589-595, doi:10.1093/bioinformatics/btp698 (2010).

11 Li, H. *et al.* The Sequence alignment/map (SAM) format and SAMtools. *Bioinformatics* **25**, 2078-2079 (2009).

12 McKenna, A. *et al.* The Genome Analysis Toolkit: a MapReduce framework for analyzing next-generation DNA sequencing data. *Genome Res* **20**, 1297-1303, doi:10.1101/gr.107524.110 (2010).

13 Gorbi, G., Moroni, F., Sei, S. & Rossi, V. Anticipatory maternal effects in two different clones of Daphnia magna in response to food shortage. *Journal of Limnology* **70**, 222-230, doi:10.3274/Jl11-70-2-05 (2011).

14 Reisser, C. M. O. *et al.* Transition from Environmental to Partial Genetic Sex Determination in Daphnia through the Evolution of a Female-Determining Incipient W Chromosome. *Mol Biol Evol* **34**, 575-588, doi:10.1093/molbev/msw251 (2017).

15 Tatarazako, N., Oda, S., Watanabe, H., Morita, M. & Iguchi, T. Juvenile hormone agonists affect the occurrence of male Daphnia. *Chemosphere* **53**, 827-833, doi:10.1016/S0045-6535(03)00761-6 (2003).

16 Toyota, K. *et al.* Methyl farnesoate synthesis is necessary for the environmental sex determination in the water flea Daphnia pulex. *J Insect Physiol* **80**, 22-30, doi:10.1016/j.jinsphys.2015.02.002 (2015).

.

**Table SD1. SNP variants in the follow up experiment**

SNP variants in the P-IT genotype and the three genotypes from Lake Ring (LRV 3 .5_15; LRV 13_2; LRV 13.5_1) as compared to the reference genome of *D. magna*. GeneID, the scaffold location (ScaffoldID), the SNP position on scaffold, the SNP variant on the reference genome of *D. magna* 2.4, and the variants in the 4 strains used in the follow up experiment are shown. The IUPAC code for amino acids is used. M: A/C; R: A/G; Y: C/T; K: G/T; S: G/C; W: A/T.

| GeneID | ScaffoldID | SNP | SNP on reference | P-IT | LRV3.5_15 | LRV13_2 | LRV13.5_1 |
| --- | --- | --- | --- | --- | --- | --- | --- |
| Dapma7bEVm001004t1 | Scaffold00027 | 3554 | A | T | A | A | A |
| Dapma7bEVm001004t1 | Scaffold00027 | 4615 | A | G | G | R | R |
| Dapma7bEVm005301t1 | Scaffold00848 | 96389 | G | C | G | G | G |
| Dapma7bEVm005301t1 | Scaffold00848 | 97167 | A | M | A | A | A |
| Dapma7bEVm002245t1 | Scaffold02003 | 35454 | G | R | G | R | R |
| Dapma7bEVm015923t3 | Scaffold02003 | 213721 | A | G | R | N | N |
| Dapma7bEVm015923t3 | Scaffold02003 | 213777 | G | R | G | G | G |
| Dapma7bEVm015923t3 | Scaffold02003 | 213793 | C | Y | C | C | C |
| Dapma7bEVm015923t3 | Scaffold02003 | 213826 | T | K | T | T | T |
| Dapma7bEVm015923t3 | Scaffold02003 | 213840 | C | T | Y | Y | Y |
| Dapma7bEVm015923t3 | Scaffold02003 | 213974 | T | G | K | K | K |
| Dapma7bEVm015923t3 | Scaffold02003 | 214099 | A | R | R | R | R |
| Dapma7bEVm015923t3 | Scaffold02003 | 214123 | T | K | K | K | K |
| Dapma7bEVm015923t3 | Scaffold02003 | 214170 | T | Y | Y | Y | Y |
| Dapma7bEVm015923t3 | Scaffold02003 | 214174 | C | A | C | C | C |
| Dapma7bEVm015923t3 | Scaffold02003 | 214254 | C | M | M | M | M |
| Dapma7bEVm015923t3 | Scaffold02003 | 214266 | C | S | S | S | S |
| Dapma7bEVm008171t1 | Scaffold02569 | 36690 | A | T | W | A | N |
| Dapma7bEVm008171t1 | Scaffold02569 | 36729 | C | A | C | C | C |
| Dapma7bEVm008171t1 | Scaffold02569 | 37124 | C | A | M | M | N |
| Dapma7bEVm008171t1 | Scaffold02569 | 37870 | A | T | W | W | N |
| Dapma7bEVm008171t1 | Scaffold02569 | 37966 | G | K | G | G | G |
| Dapma7bEVm008171t1 | Scaffold02569 | 37992 | G | R | G | G | G |
| Dapma7bEVm008171t1 | Scaffold02569 | 38011 | T | C | Y | Y | N |
| Dapma7bEVm008171t1 | Scaffold02569 | 38311 | G | R | G | R | N |
| Dapma7bEVm008171t1 | Scaffold02569 | 38315 | T | W | T | W | N |
| Dapma7bEVm008171t1 | Scaffold02569 | 39630 | T | G | G | G | G |
| Dapma7bEVm008171t1 | Scaffold02569 | 42493 | T | G | T | T | T |
| Dapma7bEVm008171t1 | Scaffold02569 | 42554 | G | R | G | G | G |
| Dapma7bEVm008171t1 | Scaffold02569 | 42696 | G | A | G | G | G |
| Dapma7bEVm008171t1 | Scaffold02569 | 42710 | T | A | T | T | T |
| Dapma7bEVm008171t1 | Scaffold02569 | 42714 | A | C | A | A | A |
| Dapma7bEVm008171t1 | Scaffold02569 | 42751 | T | C | T | T | T |
| Dapma7bEVm008171t1 | Scaffold02569 | 43000 | G | A | G | G | G |
| Dapma7bEVm008171t1 | Scaffold02569 | 43750 | A | C | M | A | N |
| Dapma7bEVm008171t1 | Scaffold02569 | 43991 | C | A | M | A | N |
| Dapma7bEVm008171t1 | Scaffold02569 | 44002 | A | G | R | G | N |
| Dapma7bEVm028519t1/Dapma7bEVm010615t1 | Scaffold02569 | 335028 | T | G | T | T | T |
| Dapma7bEVm028519t1/Dapma7bEVm010615t1 | Scaffold02569 | 336351 | C | G | C | C | C |
| Dapma7bEVm004407t1 | Scaffold02569 | 340667 | C | Y | C | C | N |
| Dapma7bEVm004407t1 | Scaffold02569 | 340708 | T | Y | T | T | N |
| Dapma7bEVm004407t1 | Scaffold02569 | 340830 | A | R | A | R | N |
| Dapma7bEVm004407t1 | Scaffold02569 | 341983 | T | K | T | T | T |
| Dapma7bEVm007919t1 | Scaffold02569 | 229366 | T | Y | T | T | T |
| Dapma7bEVm007919t1 | Scaffold02569 | 229375 | G | S | G | S | S |
| Dapma7bEVm007919t1 | Scaffold02569 | 229649 | T | A | T | A | N |
| Dapma7bEVm005463t1 | Scaffold02723 | 3857 | A | W | A | A | A |
| Dapma7bEVm001751t1 | Scaffold03156 | 4430 | G | A | A | R | N |
| Dapma7bEVm001751t1 | Scaffold03156 | 7838 | T | C | T | T | T |
| Dapma7bEVm001751t1 | Scaffold03156 | 8110 | T | C | T | Y | N |
| Dapma7bEVm001751t1 | Scaffold03156 | 8257 | C | T | T | Y | N |
| Dapma7bEVm001751t1 | Scaffold03156 | 8470 | T | A | A | W | N |
| Dapma7bEVm001751t1 | Scaffold03156 | 8542 | T | A | T | T | T |
| Dapma7bEVm015675t1 | Scaffold01036 | 709398 | A | C | M | C | N |
| Dapma7bEVm015675t1 | Scaffold01036 | 709426 | A | C | M | M | N |
| Dapma7bEVm015675t1 | Scaffold01036 | 709779 | C | T | Y | Y | N |
| Dapma7bEVm015675t1 | Scaffold01036 | 710472 | C | T | Y | Y | N |
| Dapma7bEVm015675t1 | Scaffold01036 | 710513 | G | C | S | S | N |
| Dapma7bEVm015675t1 | Scaffold01036 | 710911 | C | Y | C | C | N |
| Dapma7bEVm015675t1 | Scaffold01036 | 711065 | C | M | C | C | N |
| Dapma7bEVm015675t1 | Scaffold01036 | 711773 | T | W | T | T | T |

**Figure SD1. Genome sequence pipeline**

Pipeline used for genome data preprocessing, mapping, realignment and variant calling. For each process, the main steps and the tools used are listed.


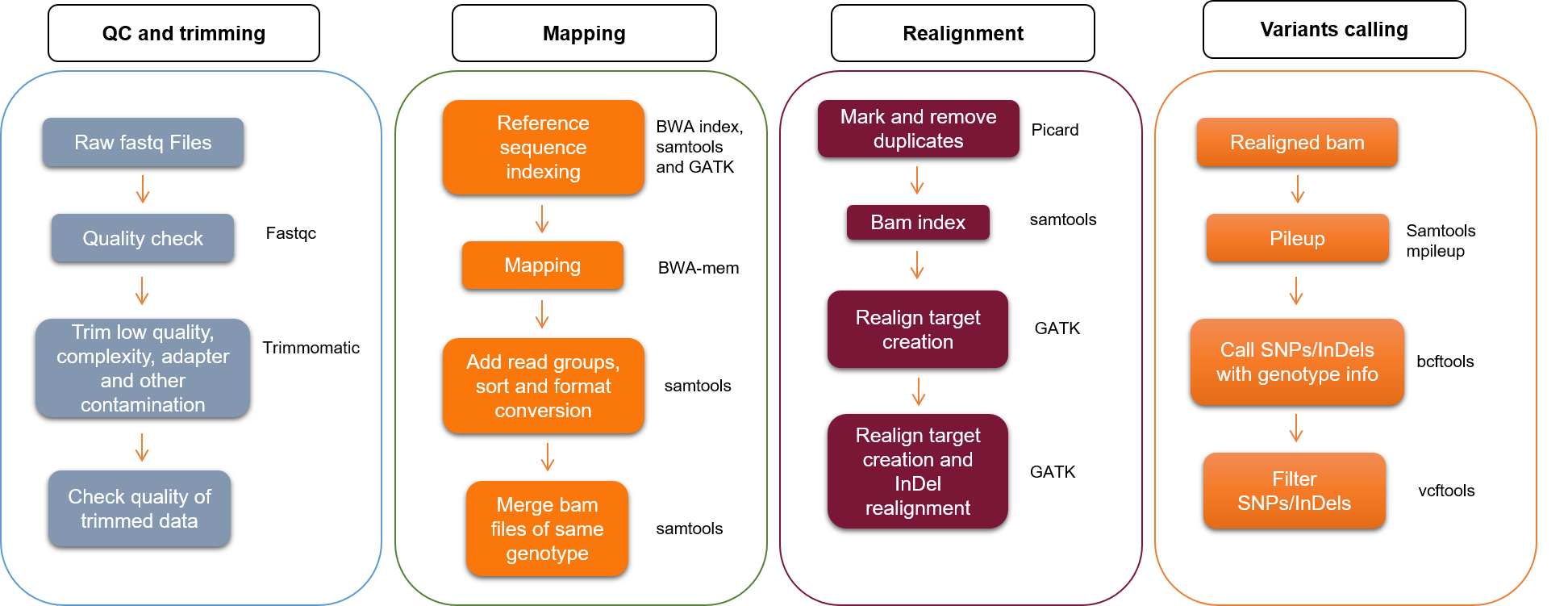


**Figure SD2. Male offspring proportion**

Percent male offspring across 8 broods in three genotypes from Lake Ring (LRV3.5_15, LRV13.2, LRV13.5_1) and in the genotype P-IT. Short photoperiod (SP) is in green; long photoperiod (LP) is in orange. Circles represent the average of 6 identical replicates ± se. CGE1 is the common garden experiment described in the main text including 30 genotypes with one replica per genotype; CGE2 is the follow up common garden experiment in which six replicates per genotype and four genotypes were used.


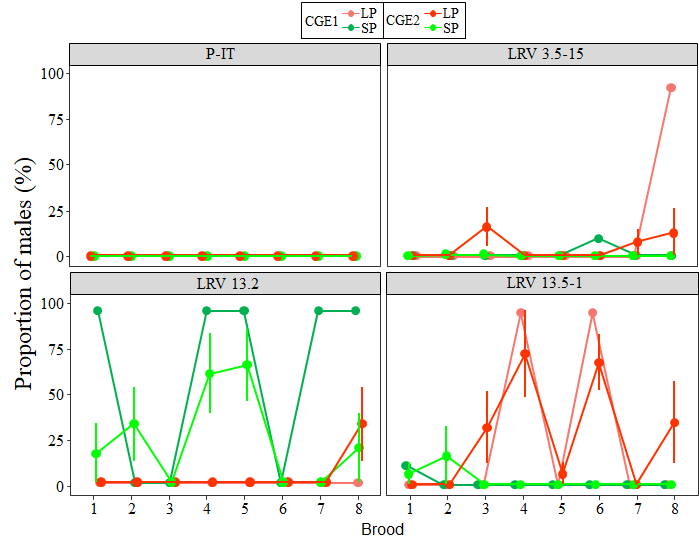

Supplement: Supplementary file 4 — SUPPORTING DATA 1 [file 41598_2019_40946_MOESM4_ESM.docx]
